# Special Issue on Refractive Surgery

**DOI:** 10.3390/jcm11030684

**Published:** 2022-01-28

**Authors:** Majid Moshirfar, Yasmyne C. Ronquillo

**Affiliations:** 1 Hoopes Vision Research Center, Draper, UT 84020, USA; yronquillo@hoopesvision.com; 2 Department of Ophthalmology and Visual Sciences, John Moran Eye Center, University of Utah, Salt Lake City, UT 84112, USA; 3 Utah Lions Eye Bank, Murray, UT 84107, USA; 4 Olivera Lab, School of Biology, University of Utah, Salt Lake City, UT 84112, USA

Laser in situ keratomileusis (LASIK) and photorefractive keratectomy (PRK), for the treatment of refractive errors, continues to evolve. LASIK, the most common refractive surgery [1], has a high success rate based on surgical outcomes and patient satisfaction. Long-term studies have shown that these procedures have a very high level of safety with rare late complications [2]. The latest refractive procedure, small incision lenticule extraction (SMILE) is at an early stage of development and rapidly gaining success rates similar to LASIK.

The Special Issue of JCM on “Refractive Surgery” is a collection of articles on refractive surgery procedures, their outcomes, complications, and alternatives. The articles will help clinicians and patients decide on procedures based on pre-op analysis and the expected or predicted outcomes. The reviewers have been constructive in their comments and the JCM editorial team was outstanding in their professionalism and support.

Basic research on laser-assisted procedures continues, with the goal of improving outcomes. The paper on “Growth Factor Rich Plasma in transepithelial photorefractive keratectomy” concludes that plasma rich in growth factor (PRGF) after transepithelial photorefractive keratectomy (TPRK) decreases immediate post-operative pain and decreases corneal re-epithelialization time [3]. The study population consisted of patients with low and moderate myopia or astigmatism in a retrospective observational study. In assessing 48 eyes of 24 patients, the mean uncorrected distance visual acuity (UDVA) was excellent at 20/20.31 LogMAR.

The research team at Hoopes Vision Research Center and medical students collaborated on outcomes-based research, as well as studying the complications and contraindications, of LASIK. A comparison of 6.0 mm vs. 6.5 mm optical zone on visual outcomes after LASIK using the WaveLIght EX500 Excimer Laser System showed that, although astigmatic correction and the post-operative angle of error were similar, outcomes tended to be worse in patients with high myopia [4]. Two study populations were matched by age and pre-operative refraction. There was no difference in post-operative uncorrected distance visual acuity (UDVA) or best corrected distance visual acuity (CDVA). Essentially, the visual outcomes are comparable to the 6.0 and 6.5 optical zones.

As with any surgical procedure, there are a few complications worth mentioning. The five-year incidence of diffuse lamellar keratitis after LASIK has fallen [5]. In previous years, a microkeratome was used for flap creation, but femtosecond (FS) lasers were recently introduced. Initial reports noted that diffuse lamellar keratitis (DLK) occurred more frequently with FS lasers than microtomes. However, this new report on 637 eyes that developed DLK noted that the incidence is approaching the microtome incidence rate. Newer, higher-frequency, lower-energy FS lasers may have contributed to the decreased initial incidence rate.

Another early complication is flap dislocation. Although the incidence of flap dislocations has decreased with the use of femtosecond lasers, they still occurred in 0.35% of 21,536 eyes that underwent laser treatments [6]. Even with dislocation, visual outcomes are excellent after repositioning, but timing is essential. The final uncorrected distance visual acuity was equal to or better than pre-LASIK CDVA in 69% of eyes. The highest risk factors for developing flap dislocation are high myopia and patient age over 50 years. With aging, the reduction in keratocyte density contributes to slower flap healing.

LASIK is not for everyone with refractive errors. There are contraindications to this procedure. There is a controversy regarding refractive surgery in patients with heritable disorders of connective tissue (HDCT) [7]. These disorders disrupt connective tissue integrity and include diseases such as osteogeneisis imperfecta and Marfan and Ehlers–Danlos Syndromes. While these disorders may lead to an increased risk of post-LASIK complications, the occurrence and severity of ocular manifestations in patients are highly variable. Those patients with minimal manifestations may be considered for LASIK. For those with more severe signs and symptoms, other options for the management of refractive errors may be more suitable. Collagen cross-linking with photorefractive keratectomy, small incision lenticule extraction (SMILE), or phakic intraocular lens implantation may be considered as viable alternatives.

Finally, phakic intraocular lenses are an alternative for those who cannot undergo LASIK or other refractive procedures. Ando and Kamiya compared phakic intraocular lens (pIOL) vaults using conventional nomogram and prediction formulas [8]. The post-operative vault was measured using anterior segment optical coherence tomography. The agreement was 50.0% when comparing the conventional nomogram and prediction formulas from the manufacturer, the NK, and the KS formulas. The achieved vault was significantly smaller than the predicted vault using the NK and KS formulas. However, for a 12.1 mm ICL size, there was no significant difference using the NK formula. With a 12.6 mm ICL size, the vault was significantly smaller with the KS formula. For a 13.2 mm size, both formulas led to significantly smaller values than the predicted vault. In conclusion, the predicted ICL vault tended to overestimate the actual ICL vault, especially when using the larger ICL size. Further refinement of the prediction formulas is necessary to improve vault accuracy.

The success rate of corneal refractive surgeries, such as LASIK, PRK, and SMILE, depends on improving pre-operative analysis and algorithms, laser platforms, and management of post-operative surprises and complications. Absolute and relative contraindications to laser refractive surgeries or other types of cornea-based surgeries must be assessed for each patient in order to offer the best alternative treatment or management, that may not be restricted to corneal refractive surgery. The management of pain and other complications may necessitate discovering biologics that avoid these and offer faster healing after surgery. The phakic IOLs are alternatives that, with improved formulas, algorithms, and design, may help those who cannot undergo cornea-based surgeries.

There will always be new horizons of treatment, such as allogenic corneal grafts, corneal on-lays, 3-D printed corneal implants, and new laser machines for refractive errors. In the not-too-distant future, we may see genetic discoveries that will change the course of diseases that lead to refractive errors. We live in exciting times of discovery, where we are able to continually assess our present methods and improve on them.

## Data Availability

Not applicable.
